# A Systematic Review of the Use of Technology to Monitor Welfare in Zoo Animals: Is There Space for Improvement?

**DOI:** 10.3390/ani11113048

**Published:** 2021-10-25

**Authors:** Alessia Diana, Marina Salas, Zjef Pereboom, Michael Mendl, Tomas Norton

**Affiliations:** 1 Department of Agronomy, Food, Natural Resources, Animals and Environment (DAFNAE), University of Padova, Viale dell’Università 16, 35020 Legnaro, Italy; 2 Zoo Antwerp Centre for Research and Conservation (CRC), Royal Zoological Society of Antwerp (RZSA), 2018 Antwerp, Belgium; marina.salas@kmda.org (M.S.); Zjef.Pereboom@kmda.org (Z.P.); 3 Centre for Behavioural Biology, School of Veterinary Science, University of Bristol, Langford House, Bristol BS40 5DU, UK; mike.mendl@bristol.ac.uk; 4 Measure, Model and Manage Bioresponses (M3 BIORES), Division Animal and Human Health Engineering, Department of Biosystems, KU Leuven, Kasteelpark Arenberg 30, B-3001 Leuven, Belgium; tomas.norton@kuleuven.be

**Keywords:** aquaria, automated monitoring, behaviour, camera, wildlife, zoological park

## Abstract

**Simple Summary:**

Ensuring appropriate animal welfare to promote wildlife conservation is a top priority of modern zoos, leading to greater effort to improve welfare monitoring approaches. However, more traditional procedures can present some limitations, while the implementation of technology might become an extra tool to comply with the need of a more efficient welfare assessment. This study aimed to summarise the available body of research on technologies used for the assessment of animal welfare in zoos. The results revealed that the majority of publications were published from 2015 onwards suggesting that this research field is still young. So far, the use of technology to assess zoo animal welfare has focused mainly on large mammals likely due to the emotional impact and interest that they have on the public and media worldwide. In addition, despite the employment of both detached and wearable sensors to assess animal welfare in zoos, implementation of algorithms to enable real-time monitoring of the animals is still scarce compared to research on farm animals. Greater application of technologies in zoo research and on more taxa should be the focus of future studies, so that another effective welfare assessment approach can be used together with more traditional procedures to improve zoo animal welfare and ultimately promote wildlife conservation.

**Abstract:**

A top priority of modern zoos is to ensure good animal welfare (AW), thus, efforts towards improving AW monitoring are increasing. Welfare assessments are performed through more traditional approaches by employing direct observations and time-consuming data collection that require trained specialists. These limitations may be overcome through automated monitoring using wearable or remotely placed sensors. However, in this fast-developing field, the level of automated AW monitoring used in zoos is unclear. Hence, the aim of this systematic literature review was to investigate research conducted on the use of technology for AW assessment in zoos with a focus on real-time automated monitoring systems. The search led to 19 publications with 18 of them published in the last six years. Studies focused on mammals (89.5%) with elephant as the most studied species followed by primates. The most used technologies were camera (52.6%) and wearable sensors (31.6%) mainly used to measure behaviour, while the use of algorithms was reported in two publications only. This research area is still young in zoos and mainly focused on large mammals. Despite an increase in publications employing automated AW monitoring in the last years, the potential for this to become an extra useful tool needs further research.

## 1. Introduction

Nowadays, one of the top priorities that modern zoos and aquaria have to fulfill is to ensure high standards of animal welfare to support and promote wildlife conservation [1,2]. In the last decades, this has led to a significant increase in the efforts made by zoos and aquaria to improve and monitor animal welfare as suggested by the Association of Zoos and Aquariums (AZA)’s Animal Welfare Committee which encourages “the development of research projects and assessment tools to advance and monitor animal welfare” [3].

To date, the variety of valuable methods used for the assessment of animal welfare ranges from monitoring physiological indicators, such as non-invasive measurement of hormones, to recording health indicators or environmental parameters and performing direct behavioural observations [4,5,6]. However, despite the useful information that zoos can gain on the welfare status of their animals, these procedures can present some limitations in terms of the amount of labor spent in manually collecting data, potential observer bias, or the requirement to use trained specialists [7,8]. In addition, if used independently, these measures may not provide a comprehensive view on the welfare state of the animals which, according to the general scientific consensus, is best achieved by combining more than one measure in order to gain an in-depth insight [5,9].

To support a proper assessment of animal welfare, all the major representative bodies for zoos and aquaria worldwide such as the AZA, the European Association of Zoos and Aquariums (EAZA), the British and Irish Association of Zoos and Aquariums (BIAZA), and the Zoo and Aquarium Association (ZAA), have decided to develop animal welfare and best care guidelines designed to assist their member institutions in pursuing and applying high standards of welfare [10,11]. However, the work is still on-going because factors such as the variety of species hosted at zoos and aquaria, the individual differences between animals, and the need to use multiple indicators within species, pose a challenge to the development of tailored systematic methods for the assessment of the animals. This in turn, leads to a lack of routine welfare monitoring systems [12]. To date, only a few species-specific tools have been developed and validated including those for elephants [13,14], bottlenose dolphins (*Tursiops truncatus*) [15], and dorcas gazelles (*Gazella dorcas*) [16]. Thus, more effort and collaboration are needed by the entire international zoo community for the development of species-specific welfare assessment protocols following the example of the Welfare Quality^®^ project launched in 2004 by the European Commission to evaluate animal welfare in farm species [17]. This highlights the importance of sharing approaches and expertise between research groups working on captive species (i.e., farm, lab, and zoo animals) [18].

Such time-consuming procedures and their associated limitations clearly emphasise the need for the development and application of more cost-effective and yet non-invasive methods for the assessment of welfare in captive wild animals. So far, the incorporation of technology into zoo research has been mainly applied to promote good welfare via enrichment challenges [19,20] such as touchscreen computers, computer-controlled feeding systems, interactive projections, or computer-based cognitive tasks [21,22,23,24]. Indeed, providing animals with increased choices and better control of their environment may help to improve their welfare state [25,26]. However, to comply with the need of a more efficient welfare assessment, a further implementation of technology as an additional tool in zoo research might be the right direction to fill some of the gaps previously discussed. Furthermore, in the last few years, technology has become more affordable and accessible and shown great potential for monitoring farm animal welfare in the field of precision livestock farming (PLF) [27]. By continuously and automatically monitoring the status of the animals and their environment in real time, PLF is a well-recognised tool that, via an early warning, permits the farmers to promptly intervene to solve welfare issues in their herds. To do so, sensor technologies and computational analysis are combined to provide insight into behavioural and physiological functioning of the animals in a non-invasive way [8,28], while also offering a more accurate picture of their overall wellbeing compared to the snapshot furnished by more traditional assessment methods.

Clearly, there seems to be space for improvement in the use of technology in zoo research as real-time bioresponse modelling has the potential to become an additional effective solution to automating welfare assessment of zoo species. Moreover, given that the field of animal monitoring technology is fast developing, the extent to which it is being adopted for zoo animal welfare monitoring is still unclear. Therefore, with the current systematic review, we aimed to summarise the available body of research on the use of technology for the assessment of animal welfare by measuring animal-based and environmental parameters in zoos and aquaria (hereafter zoos), with a focus on sensor technologies used to continuously and automatically monitor welfare in real-time.

## 2. Materials and Methods

### 2.1. Systematic Literature Search and Strategy Applied

A systematic literature search was performed by a single researcher in June 2021 using the databases PubMed and Web of Science. The search included English-only publications with no limitation for publication year and accounted initially for all types of documents (e.g., articles, reviews, and books).

Prior to the start of the technical search, a concept map and the associated keywords, presented in Table 1, were defined and agreed upon by two researchers. The concept map consists of three columns which represent the three concepts used for the search: (1) animals studied, i.e., zoo animals; (2) the method used, i.e., use of technology; (3) the subject investigated, i.e., animal welfare. Each concept was first searched individually, and then they were combined in a single search. The first systematic search was conducted in the database “PubMed” using the field Title/Abstract [*tiab*] added to each keyword because the more generic field Textwords [*tw*] brought up too many false results. The second systematic search was conducted in the database “Web of Science” using the fields Title only [*TI*] and Abstract only [*AB*] placed before the list of all keywords associated with each concept because the more generic field Topic [*TS*] brought up too many false results. The two lists were, then, combined by the Boolean “OR”. For both systematic searches, each keyword within each concept of the map was separated by the Boolean “OR” while the concepts were separated by the Boolean “AND”.

### 2.2. Eligibility Criteria and Screening

Publications obtained from the systematic literature search were imported to Mendeley (Mendeley Desktop, version 1803, Elsevier, Amsterdam, The Netherlands) to systematically perform the selection of relevant papers. In order to finalise the number of publications to be used for the systematic review, the following steps were applied by a single researcher according to the PRISMA guidelines [29]: First, all duplicates were excluded. Second, the abstract of each publication was evaluated according to the following exclusion criteria: (1) not involving the use of technology to assess animal welfare in zoo animals; (2) not concerning animal welfare and/or use of technology of zoo species, i.e., publications regarding the use of technology on animals in nature/lab/sanctuaries/research centres were excluded; (3) not concerning studies on animal species; (4) use of technology to improve animal welfare (e.g., as an environmental enrichment) but not to assess animal welfare; (5) is a conference abstract/paper; (6) not a peer-reviewed publication. Third, the full text of all publications falling within the above criteria were extracted for an in-depth screening to establish the final number of publications to include in the systematic literature.

Finally, the full text of all publications considered relevant for the systematic literature analysis were screened by two researchers (A.D. and M.S.) independently. Then, the two independent analyses were compared and if there were uncertainties during the screening process, the publications were further checked by the two researchers in order to reach an agreement. For each publication, the following information was noted: (1) title; (2) journal; (3) year of publication; (4) animal species studied; (5) country where the study was conducted; (6) country of the affiliation(s) of the first author; (7) type of technology used to measure the parameters classified as wearable sensors (e.g., GPS, RFID, and accelerometer), camera, microphone, and other (e.g., ECG and ultrasonography); 8) type of parameters measured (i.e., behavioural, physical/physiological, and environmental). The raw data are available as Supplementary Material (Table S1).

## 3. Results

### 3.1. Systematic Literature Search and Strategy

The systematic search strategy led to 753 publications from the database “PubMed” and 1353 publications from the database “Web of Science” for a total of 2106 publications. Out of these, 595 publications were duplicates. The abstracts of the 1511 remaining publications were screened for eligibility according to the exclusion criteria and of those, 19 publications were considered to be of relevance for the systematic review. Out of these publications, 2 were reviews and 17 were research studies. Of those, 1 publication was a retrospective study, 1 publication was a case study, and 15 publications were experimental studies.

### 3.2. Characteristics of the Publications

**Figure 1:**
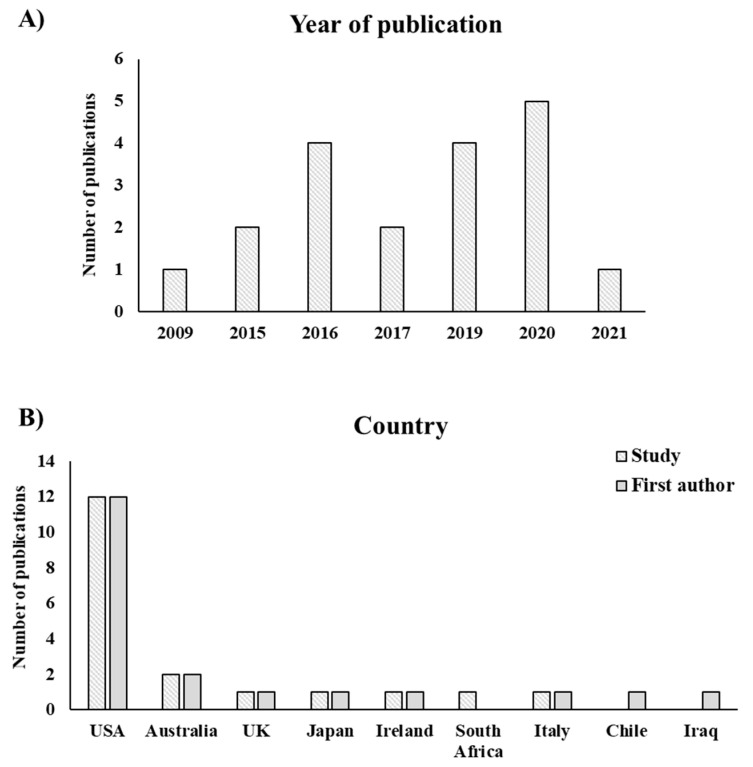
(**A**) Number of publications by year of publication; (**B**) country of the study and country of the first author’s affiliation. Publications can have more than one country for the first author.

Figure 1 and Table 2 and Table 3 provide the general characteristics of the 19 publications obtained from the search. Information on year of publication, journal, animal species studied, country of the study, and country of the first author main affiliation are described. The earliest publication was published in 2009, while all the remaining 18 publications were published starting from 2015 onward with 66.7% of them published between 2015 and 2019 and 33.3% of them only in the last two years (i.e., 2020–2021).

The most studied animal species was elephant (36.8%) followed by primate species (e.g., gorilla, orangutan, and lemur – 21%) with 21% of the publications investigating more than one species. Specifically, mammals – class: Mammalia – was the group mainly represented in the publications (89.5%) followed by birds – class: Aves (21%). The leading peer-reviewed journals with the highest percentage of publications were *Zoo Biology* (31.5%) and *Animals* (26.1%), representing together more than half of the total publications. The most represented country of the study and first author main affiliation was USA (63.1%) followed by Australia (10.5%).

### 3.3. Type of Technology and Parameters

An overview of sensor technologies applied to measure animal-based and environmental parameters to assess animal welfare in zoo animals can be found in Table 4. The majority of the publications (*n* = 17) used technology to measure behavioural (63.1%) and physical/physiological (31.6%) parameters, while only 3 publications used and/or reviewed sensor technology to measure environmental parameters such as water/air temperature and sound/noise level. Three publications measured more than one type of parameter. Fifty-two percent of the publications used a camera as the technology to assess animal welfare, followed by wearable sensors (31.6%) such as accelerometers, GPS, and RFID tags. There were only 2 publications that reported the use and/or development of an algorithm to monitor animal welfare.

## 4. Discussion

### 4.1. Automated Animal Welfare Monitoring: A Young Field in Zoo Research

The aim of this systematic review was to provide an overview of the current state of research on the use of technology to monitor animal welfare in zoo animals, and specifically, on technologies used to automatically and continuously monitor animal-based or environmental parameters in real-time. This search is fundamental to evaluate the progress of this fast-developing field since little information is available on animals hosted in zoos compared to their counterparts kept on-farms. The small number of publications identified through the systematic search (*n* = 19), which compared to some reviews on farm species such as pigs (*n* = 101) [49] and poultry (*n* = 264) [50] are minimal, confirmed our assumption. Moreover, the majority were published from 2015 onwards with a third of the total publications in the last two years. This finding highlights how the employment of this research approach is still at an early stage in zoo research, and that despite a recent increase in publications, the potential for automated animal welfare monitoring to become a useful tool in zoos needs further investigation.

A trend towards English-speaking countries as the main location and first author’s institution of the study emerged from the data, in particular, more than half of the studies were carried out in the US. It is important to note that only papers published in English, the most used language in peer-reviewed journals, were included in this systematic review, thus, it is likely that inclusion of non-English speaker publications, especially with regards to the variety of languages spoken in the EU, may have led to different results. In addition, when considering the single country, the US holds the most numerous accredited zoos worldwide [51,52] which are also among the largest. Another aspect to take into account is the difference between US and EU in legislation protecting the welfare of captive animals. The policy applied in the EU regarding the transparency/privacy of sensitive data are likely to be more strict compared to that in the US, thus requiring greater efforts spent in bureaucracy and a slowing-down of research procedures [53,54]. Overall, this might partly explain why the majority of publications were from US zoos and institutions.

Mammals were the most studied group of animals with Asian/African elephants as the main species investigated followed by primates. This result is expected because large mammals are among the most studied animals in zoos not only for their biological importance but also because of the emotional impact and interest that they have on the public and media worldwide [12,55,56]. They are considered to be “ambassadors” of their own species in the wild making the need of maintaining high standards of welfare a top priority of modern zoos [57]. This likely explains the larger body of research available so far on the methods - i.e., employing either a more traditional or sensor-based technology approach - used to assess and improve animal welfare in large mammals compared to other species hosted in zoos. However, this should not reduce the interest/need for investigating other taxa which deserve as much attention as mammals in promoting wildlife conservation and good welfare. For instance, reptiles and amphibians have always been considered a minor priority in zoos because their welfare was seen as less affected by captivity than mammals or birds, possibly because they are regarded as less likely to be sentient, and hence conscious experience of negative (and positive) affective states than “feeling” mammals [12,58,59]. Thus, despite these taxa being highly threatened in the wild [60], further work is still required to expand the taxonomic focus of animal welfare monitoring.

The journals selected for publication are those mostly concerned with all aspects of wildlife, conservation, and animal welfare such as *Zoo Biology*, *Journal of Zoo* and *Aquarium Research*, and *Animals* and all with an open-access publication option. The latter is specifically of great importance because it can contribute to easily disseminate the findings of this novel field in the zoo setting. However, the employment of more technology field-specific journals is still limited (i.e., we found only one publication in the journal *Sensors* [37]), likely due to the fact that these journals are more focused on recent advances in agriculture, livestock farming, and land sustainability where the application of automated monitoring systems is clearly greater. Thus, we suggest that a more interdisciplinary approach with regard to the choice of the most suitable journal is needed for zoo research on automated monitoring systems.

### 4.2. Use of Technology in Zoos and Aquaria to Monitor Animal Welfare

Individual-level sensors (e.g., accelerometers, GPS, and RFID) were used to monitor animal welfare in zoos as much as those employed for a group-level monitoring approach (e.g., cameras). This is in contrast with the trend reported in farm research. For instance, Larsen et al. [49], in their review on pigs, showed that over 80% of the publications used detached sensors (e.g., microphones and cameras) because this allowed the monitoring of the entire pen without disturbing and/or interfering with the animals. In addition, the use of individual-level sensors was not considered a cost-effective tool for farmers. The difference in the most used sensors between zoo and farm animals may also be justified by the lower number of animals to be monitored at the zoo and the different housing conditions provided to the two categories of captive animals. If the aim is to monitor the welfare status of each animal, a camera or microphone - which are likely to have a limited area of action - can be less feasible to apply due to the wide space available and the natural-like design of the zoo enclosure compared to the farm pen. Indeed, one of the core aspects to maintain a good welfare status is to provide an environment that allows the animals to hide themselves from visitors when needed [61,62]. Therefore, a more suitable approach to monitor animal welfare indicators combined with a lower number of individuals to monitor, can help to explain the high use of individual-level sensors in zoo research compared to farm studies.

The potential to advance the monitoring of zoo animal welfare by the use of technology was reported throughout the papers included in the current review. Although technology can integrate the current approaches applied in zoos for animal welfare monitoring and assessment, scientists are still far from taking full advantage of it [35]. For instance, when collecting physiological indicators of welfare such as heart and breathing rate or body temperature, the use of devices such as digital [37] and IR thermal cameras [39] or mobile ECG monitors programmed with algorithms [46] had the advantage that animals did not need to be anesthetized nor to carry over equipment which can be stressful and invasive procedures. A study on gorillas also suggested that IR thermal cameras may have the potential to investigate the emotional response of the animals; however, the authors stated that the application of this device in the absence of other related physiological/behavioural measures needs further validation [38]. Technology can also be promising to gather additional and more accurate notions on health and welfare parameters that a more traditional assessment can miss at times, thus allowing for further investigations of poorly studied welfare aspects [30,36,43,47]. In a study on cheetahs where both direct observation and cameras were employed to collect behavioural data [33], the latter were able to identify some behaviours that the traditional approach did not detect. Indeed, cameras may record animals in areas of difficult access for direct observation and avoid the presence of the observer. In addition, anklets equipped with accelerometer and GPS loggers helped to investigate elephants’ recumbence behaviour on a large scale (40 zoos) and for 24 h/day [42]. Similarly, using sensor technology such as microphones and data-loggers to continuously measure environmental parameters can provide extra information on how they may influence animal welfare. A better understanding of animals by integrating both approaches, as seen in studies on hippos [48] and giant anteaters [40], can ultimately be useful for management decisions. The use of wearable sensors such as GPS collars and RFID tags was also found promising by the authors in determining social relationships in elephants [41] and behavioural swimming patterns in penguins [45] by overcoming possible disadvantages of traditional methods like the observer fatigue, visual obstruction, and lack of identifying certain behaviours. Similar conclusions were reported in other studies [30,31,32,34] where the use of multiple technologies (e.g., IR cameras, CCTV, and camera traps) contributed to minimise time and resources usually necessary for continuous monitoring, while also providing much insight into the animal behaviour associated with weather conditions and time of the day when such observations are difficult to achieve (i.e., nocturnal activities).

Another comparison worthy to note is the increased use of automated monitoring systems of animals under natural field conditions as a valuable method to investigate their movements and behaviour without interference [63,64]. The ecological necessity of tracking animals in the wild has always been of high importance due to conservation concerns. Recently, this has led to significant use of technology to monitor wildlife. From GPS collars, radio-telemetry to remote sensing, the rapid advance of the use of technology in the wild has a longer research history compared to the zoo environment [65,66,67]. Last but not least, there are also studies on the implementation of algorithms to enable real-time monitoring of the animals for conservation-related projects - such as the management of threatened species [68,69] - or to develop early warning systems to deal with issues such as the human-elephant conflict [70,71]. In the current review, the monitoring of zoo animal welfare in real-time by employing algorithms was reported in two publications only [44,46] - both reporting how promising this tool could be to provide objective and quantifiable measures of welfare parameters - which emphasises that further research on machine learning methods is much needed among the zoo community. Hence, complementing traditional approaches with automated welfare monitoring can yield to greater beneficial to the assessment of zoo animal welfare as already observed in farm animal research. Indeed, this will permit not only better investigations of some aspects of welfare assessment that are still poorly understood - such as the role of anticipatory behaviour [72] - by combining multiple indicators at once (e.g., behavioural, physiological and cognitive responses), but also continuous and automatic monitoring of the welfare status of the animals to provide a warning when something goes wrong.

Nevertheless, in spite of the aforesaid potential benefits that technology and automated monitoring systems can add to the assessment of zoo animal welfare, there are also some disadvantages that should not be underestimated. Among others, there is the cost factor that, although more affordable than in the past years [27], may still be prohibitive to sustain for some zoos [19,20]. Application of the field of machine learning may also be out of the reach for many zoos, emphasising the need for strong collaborations with research groups that hold expertise in this area. Last but not least, when individual-level sensors are used, the animals have to, first, go through training procedures and a habituation process to ensure that these devices are not disturbing them or influencing their behaviour.

### 4.3. Limitations of the Study

A potential limitation of this study can be related to the search methodology and the associated final number of publications. We are aware that not all publications in-line with the objective of the current work were likely identified by the systematic literature search. For instance, publications published in journals that were not peer-reviewed or those not included in PubMed and Web of Science [73]. Indeed, we agreed to not include gray literature in this review because we considered the search on global citation databases such as Web of Science, PubMed and similar, which are commonly used by the international scientific community when doing a search of literature [74,75], to be the most suitable approach to apply for the current review paper. Moreover, due to the link between the indicators (i.e., behavioural, physiological, or environmental-based) used to monitor animal welfare and the meaning itself of “animal welfare assessment”, we considered the keywords “welfare” and similar as the best representative/inclusive terms for the concept “animal welfare” defined in the concept map. However, despite this allowing us to narrow down the search, it may have limited the detection of publications where the term welfare and similar words were not included in the title or abstract, emphasising the importance of keywords during a systematic search.

## 5. Conclusions

Although several review papers clearly stated the need for improvement in the use of automated monitoring technology as an additional tool to advance animal welfare in zoos [35,76], specifically with regards to the development of algorithms to enable real-time welfare monitoring, the body of research available so far is scarce. Indeed, this research area is still young in zoos, as the majority of publications were published from 2015 onwards, and it is mainly focused on large mammals likely due to the emotional impact and interest that they have on the public and media worldwide. Greater interest for investigating more taxa, which deserve as much attention as mammals in promoting wildlife conservation and good welfare, should also be the focus of future studies. An increase in publications employing automated animal welfare monitoring has been observed in recent years, nevertheless, the potential for this fast-developing field to become a widespread additional useful tool in the zoo setting needs further research.

## Figures and Tables

**Table 1:** The three concepts and the associated keywords used for the systematic literature search.

	Concepts	
1. Zoo Animals	2. Use of Technology	3. Animal Welfare
Zoo	“Continuous monitor *”	Wellbeing
Zoos	Automat *	Well-being
Aquarium	Algorithm *	Welfare
“Captive animal *”	Sensor *	
Zoolog *	Analysis *	
Primate *	Radio *	
“Wildlife park *”	Video *	
Reptile *	Image *	
Amphibian *	Sound *	
Ungulate *	Prediction *	
Elephant *	Accelerometer *	
Bear *	Technolog *	
Bird *	Microphone *	
“Big cat”	Camera *	
“Wild cat”	Remote *	
Felid *	Digital *	
Canid *	Computer *	
Mammal *	“Early warning”	
Prosimian *		
Monkey *		
Equid *		
Seal *		
“Marine mammal *”		

Terms in quotation marks (“) are considered in the search as one word whereas the asterisk (*) is used to indicate the request of searching for all the terms that begin with that word (e.g., plurals).

**Table 2:** Number and percentage of publications by group and studied species. Publications can have more than one studied species.

Group Studied	Species Studied	*n*	%
	Elephant (Asian/African)	7	36.8
	Primates species	4	21.0
	Koala	3	15.8
	Marine mammals	3	15.8
Mammals	Felid species	2	10.5
(*n* = 17)	Ursidae species	2	10.5
	Hippopotamus	1	5.3
	Giraffe	1	5.3
	Giant anteater	1	5.3
	Kangaroo	1	5.3
	Alpaca	1	5.3
Birds	Penguin	3	15.8
(*n* = 4)	Carmine bee-eater	1	5.3

**Table 3:** Number and percentage of publications by peer-reviewed journal.

Peer-Reviewed Journal *	*n*	%
*Zoo Biology*	6	31.5
*Animals*	5	26.1
*Sensors*	1	5.3
*Plos One*	1	5.3
*Animal Welfare*	1	5.3
*Marine Mammal Science*	1	5.3
AABS	1	5.3
JZAR	1	5.3
JAAWS	1	5.3
JVB-CAR	1	5.3

* AABS, *Applied Animal Behaviour Science*; JZAR, *Journal of Zoo and Aquarium Research*; JAAWS, *Journal of Applied Animal Welfare Science*; JVB-CAR, *Journal of Veterinary Behavior-Clinical Applications and Research*.

**Table 4:** Number of publications by type of technology used for the study and type of parameter investigated. Publications can have more than one technology used and parameter investigated.

Type of Technology	Type of Parameter	Citation
	Behavioural (*n* = 7)	[30,31,32,33,34,35,36]
Camera (*n* = 10)	Physical/physiological (*n* = 4)	[35,37,38,39]
	Environmental (*n* = 1)	[35]
Microphone	Environmental (*n* = 1)	[40]
(*n* = 2)	Behavioural/physiological (*n* = 1)	[35]
Wearable sensors	Behavioural (*n* = 6)	[35,41,42,43,44,45]
(*n* = 6)	Physical/physiological (*n* = 1)	[35]
	Behavioural (*n* = 2)	[31,35]
Other (*n* = 5)	Physical/physiological (*n* = 3)	[35,46,47]
	Environmental (*n* = 2)	[35,48]
Algorithm	Behavioural (*n* = 1)	[44]
(*n* = 2)	Physical/physiological (*n* = 1)	[46]

## Data Availability

The raw data presented in this study are available in Supplementary Material Table S1.

## Supplementary Materials

The following are available online at https://www.mdpi.com/article/10.3390/ani11113048/s1, Excel file Table S1: Raw data of the final publications selected for the systematic review.
